# Stress induced obesity: lessons from rodent models of stress

**DOI:** 10.3389/fnins.2013.00130

**Published:** 2013-07-24

**Authors:** Zachary R. Patterson, Alfonso Abizaid

**Affiliations:** Department of Neuroscience, Carleton UniversityOttawa, ON, Canada

**Keywords:** stress, obesity, animal models, hormones, feeding behavior, hypothalamus

## Abstract

Stress was once defined as the non-specific result of the body to any demand or challenge to homeostasis. A more current view of stress is the behavioral and physiological responses generated in the face of, or in anticipation of, a perceived threat. The stress response involves activation of the sympathetic nervous system and recruitment of the hypothalamic-pituitary-adrenal (HPA) axis. When an organism encounters a stressor (social, physical, etc.), these endogenous stress systems are stimulated in order to generate a fight-or-flight response, and manage the stressful situation. As such, an organism is forced to liberate energy resources in attempt to meet the energetic demands posed by the stressor. A change in the energy homeostatic balance is thus required to exploit an appropriate resource and deliver useable energy to the target muscles and tissues involved in the stress response. Acutely, this change in energy homeostasis and the liberation of energy is considered advantageous, as it is required for the survival of the organism. However, when an organism is subjected to a prolonged stressor, as is the case during chronic stress, a continuous irregularity in energy homeostasis is considered detrimental and may lead to the development of metabolic disturbances such as cardiovascular disease, type II diabetes mellitus and obesity. This concept has been studied extensively using animal models, and the neurobiological underpinnings of stress induced metabolic disorders are beginning to surface. However, different animal models of stress continue to produce divergent metabolic phenotypes wherein some animals become anorexic and lose body mass while others increase food intake and body mass and become vulnerable to the development of metabolic disturbances. It remains unclear exactly what factors associated with stress models can be used to predict the metabolic outcome of the organism. This review will explore a variety of rodent stress models and discuss the elements that influence the metabolic outcome in order to further extend our understanding of stress-induced obesity.

## Introduction

It is now generally accepted that obesity has reached epidemic proportions. To some medical professionals, obesity is considered the leading preventable cause of death worldwide. More worrisome is the fact that the prevalence of obesity has drastically increased in the past 30 years, where ~10% of the population was considered overweight in the 1970's, a number that has now risen to over 35% in the United States (Flegal et al., 2010, 2012). This estimate is accompanied by a wealth of statistics demonstrating that individuals who are overweight or obese have a much higher susceptibility to diseases such as Type II diabetes, cardiovascular disease and metabolic syndrome. In fact, some studies show that life expectancy decreases significantly as a function of weight gain, and that individuals suffering from obesity die up to a decade sooner than those of a normal weight (Peeters et al., 2003). For this reason, obesity is one of the most pressing health issues seen in the twenty-first century.

It is clear that there is a strong genetic predisposition to obesity and metabolic disorders. Nevertheless, environmental and social factors influence amounts of food consumed or energy spent and ultimately impact phenotype. Arguably, stress may be the most important environmental factor affecting metabolism and feeding behavior to promote obesity. Indeed, there is an association between adiposity, particularly abdominal adiposity, and psychological distress (Raikkonen et al., 1996; Chrousos and Gold, 1998; Bjorntorp, 2001). Furthermore, and in parallel with the increasing rates of obesity, subjects from industrialized communities report higher levels of workplace stress and sleep deprivation in recent years relative to the past (Bjorntorp, 2001; Caruso, 2006; Grosch et al., 2006).

The physiological and behavioral responses to stress are necessary for survival. During an acute stress response, activation of the hypothalamic-pituitary-adrenal (HPA) axis entails a cascade of events that begins with the release of corticotropin-releasing factor (CRF) from specialized neurons in the paraventricular nucleus of the hypothalamus (PVN) into the portal blood neighboring the anterior pituitary gland. CRF secretion in turn leads to the synthesis and release of adrenocorticotropic hormone (ACTH) from the anterior pituitary into the general circulation, which ultimately stimulates the release of glucocorticoids from the adrenal cortex. ACTH exerts negative feedback control over CRF at the level of the hypothalamus and, along with CRF, orchestrates the body's response to a homeostatic challenge (Aguilera, 1994). Glucocorticoid release from the adrenal glands is the final output stage of the HPA axis, which represents, amongst other things, an organism's attempt to mobilize energy stores to fuel the brain, skeletal and cardiac muscles.

The primary glucocorticoids secreted in response to stress are cortisol and corticosterone in humans and rodents, respectively. In many tissues, glucocorticoids serve an opposing function to insulin's attempt at storing energy, in part by suppressing insulin secretion (Dinneen et al., 1993). By inducing a transient state of insulin resistance, glucocorticoids restrain hepatic glucose production and regulate glucose delivery to peripheral tissue thereby producing an acute state of hyperglycemia (Rizza et al., 1982; Mangos et al., 2000). The peripheral tissues involved in the stress response use the acute hyperglycemic environment to meet the energetic demands of the stressor.

The aforementioned secretion of CRF and subsequent release of glucocorticoids is under the control of a finely tuned negative feedback loop, wherein elevated circulating glucocorticoids travel back to the hypothalamus (Jones et al., 1977; Di et al., 2003, 2005), hippocampus (Sapolsky et al., 1984, 2000), medial prefrontal cortex (Diorio et al., 1993; Radley et al., 2008) and pituitary gland (Mahmoud et al., 1984; Cole et al., 2000) to inhibit further stimulation of the HPA axis (McEwen, 2007; Ulrich-Lai and Herman, 2009). At the level of the hypothalamus, glucocorticoids have been shown to cause rapid suppression of excitatory synaptic inputs to CRF neurons within the PVN (Di et al., 2003), an effect that is thought to be mediated by the retrograde action of endocannabinoids on upstream glutamate neurons (Di et al., 2005). In contrast, glucocorticoid feedback at the level of the hippocampus increases excitatory post-synaptic potentials via modulation of membrane-bound mineralocorticoid receptors (Olijslagers et al., 2008). Rapid excitation of efferent hippocampal neurons, in turn, leads to the activation of inhibitory GABAergic input onto the neuroendocrine PVN neurons (Boudaba et al., 1996; Herman et al., 2002) and is therefore implicated in the negative feedback regulation of the HPA axis (Furay et al., 2008). Furthermore, the medial prefrontal cortex (mPFC) is also responsive to stress-induced secretion of glucocorticoids and is capable of directly modulating the activity of secretory PVN neurons (Radley et al., 2008). Injections of corticosterone (CORT) into the mPFC results in significant reductions of CRF and ACTH release following restraint stress in rats (Diorio et al., 1993), demonstrating a role for the mPFC in the regulation of HPA axis activity. After answering the energetic demands posed by a stressor, the negative feedback loop of the HPA axis ensures an efficient return to homeostatic balance following cessation of the stressor and it is therefore considered a mediator of allostasis.

The acute response to stress is one that is both necessary and beneficial because it mediates the behavioral and physiological adaptations necessary for homeostatic maintenance under stressful conditions. Protracted stressors, on the other hand, lead to an excessive energetic burden resulting in long lasting physiological changes, a process referred to as allostatic overload (McEwen, 2004). Failure to adjust to the energetic demands posed by this overload, or failure to reduce the stressor itself, may lead to negative health consequences, both metabolic and psychiatric. Increasing rates of chronic daily stress, regardless of their origin, in combination with increased availability of foods high in calories creates an environment conducive to the development of obesity and other metabolic disturbances. In turn, the rising incidence of obesity is detrimental to health care as it exerts a financial burden to taxpayers while saturating medical services that would otherwise be available. The interaction between neural circuits involved in regulating energy homeostasis and body composition with the physiological responses to stressors has therefore become pertinent and clinically relevant. In attempt to shed light on this matter, scientists must turn to one of the most widely used tools for studying the molecular underpinnings of disease, which is the use of animal models. The present review aims to investigate the use of animal models in the study of stress and to highlight some of the factors that contribute to the metabolic outcome of these various stress paradigms.

## Stress, eating and energy homeostasis

Stress influences feeding behaviors and energy homeostasis in both humans and rodents. However, the neural mechanisms that are recruited by the stress response are complex and involve a number of circuits that include hypothalamic networks regulating energy balance, brain stem networks regulating visceral function, sympathetic and parasympathetic mechanisms, midbrain nuclei involved in reward and arousal, and corticolimbic structures that mediate emotional and cognitive aspects related to both stress and food intake. Furthermore, stress produces dramatic endocrine and neuroendocrine responses that in themselves affect energy balance and food intake. Among these, the best-characterized responses are the activation of the sympathetic nervous system and that of the HPA axis. The former is elicited to generate rapid physiological responses including increased heart rate and respiration, glucose uptake in muscle and decreased visceral function, all important for generating a fight or flight response. Subsequent activation of the HPA axis, as previously discussed, represents a hormonal cascade that culminates in the secretion of glucocorticoids. These steroid hormones have a multitude of effects, both in the periphery and the brain that among other things alter metabolism, promote appetite and increase the locomotor activities associated with food seeking behaviors in rodents (McEwen, 2004).

Numerous studies have shown that stress modulates metabolism, but these effects are not always consistent and can range from marked decreases in both body weight and food intake, to increases in body weight and calorie consumption (Dallman et al., 2005; Depke et al., 2008; Nieuwenhuizen and Rutters, 2008). For example, acute psychogenic or systemic stressors results in a rapid decline of food intake and the successive breakdown of nutrient stores (Depke et al., 2008). Similarly, exposure to chronic stressors can also lead to decreased body weight and food intake (Torres and Nowson, 2007; Depke et al., 2008). While these animals appear to lose weight, they do by using nutrients derived from tissues other than fat in spite of decreasing their body weight (Akana et al., 1999; Depke et al., 2008). Metabolically, these animals show altered glucose regulation, an early indicator of insulin resistance (Black, 2006).

Interestingly repeated exposure to psychogenic stressors, in particular social stressors, generates increases in body weight, adiposity, and in the intake of high calorie “comfort” meals in a number of species including non-human and human primates (Pecoraro et al., 2004; Dallman et al., 2005; Tamashiro et al., 2007a; Coccurello et al., 2008; O'Connor et al., 2008; Wilson et al., 2008). These data are of particular relevance considering that in human populations stress is highly associated with social interactions (Tamashiro et al., 2007a,b; Michaud et al., 2008; O'Connor et al., 2008; Wilson et al., 2008). The mechanisms underlying metabolic changes in response to social stressors have commonly been associated with the negative consequences of repeated stimulation of the HPA axis (Anisman and Matheson, 2005). In the case of obesity, elevated levels of glucocorticoids appear to be associated with abdominal adiposity (Black, 2006). Indeed, clinical conditions characterized by high cortisol levels, as seen in patients with Cushing's or Prader–Willi syndrome, are also associated with abdominal obesity and type II diabetes (Nieuwenhuizen and Rutters, 2008; Weaver, 2008). In contrast, removal of the adrenal glands in experimental animals results in decreased body weight and food intake that can be restored by corticosterone replacement (Saito and Bray, 1984; Pralong et al., 1993; Makimura et al., 2000).

Finally stress also affects psychological processes that could lead to the preference of foods that have a comfort value. For instance, some stressors have been associated with a loss of control over food intake and an increased drive to eat highly palatable non-nutritious foods (Groesz et al., 2012). This active seeking of highly palatable foods is thought to be a form of self-medication for both humans and rodents, wherein HPA axis activation is down regulated in response to the rewarding properties of such foods ultimately creating a preference, and potentially cravings, for these foods (Dallman et al., 2003, 2005; Pecoraro et al., 2004; Tomiyama et al., 2011). Interestingly, while all stressors are known to simulate the HPA axis, there is a documented, bi-directional effect on food intake. Physical stress, for example, may cause a reduction in food intake (Popper et al., 1989) while psychological stress can increase or decrease food intake (Huhman, 2006).

### Regulation of food intake and energy balance: A look inside the brain

To understand how stress influences metabolism and feeding we first need to consider how feeding and metabolism are regulated and then how stress can influence these mechanisms. Within the brain there exists a very complex organization of nuclei that collectively control food intake and energy homeostasis. For a more comprehensive review on the many brain circuits regulating energy homeostasis and the integration of afferent metabolic signals see Horvath et al, (2004) Abizaid and Horvath (2008).

Traditionally, the hypothalamus is considered the focal point for processing and integrating both afferent and efferent metabolic information (Elmquist, 2000; Schwartz et al., 2000; Dhillo and Bloom, 2001; Cummings and Shannon, 2003). Within the hypothalamus, these nuclei can be roughly divided into two distinct groups based on their tendencies to promote (lateral hypothalamus, LH) or inhibit food intake (ventromedial nucleus and paraventricular nucleus; VMH and PVN, respectively). Furthermore, at the base of the hypothalamus the arcuate nucleus (ARC) lies within an area that is less protected by the blood brain barrier and is thus capable of detecting peripherally circulating metabolic signals (Ciofi, 2011; Morita and Miyata, 2012, 2013). With this real estate, and the expression of both orexigenic and anorectic peptides, the ARC is thought to play an intimate role in the regulation of food intake and energy homeostasis (Schwartz et al., 2000; Cone et al., 2001; Cowley et al., 2001, 2003). The influence of the ARC on feeding behaviors is mediated by a sub-set of first-order neurons that either co-express the orexigenic transcripts neuropeptide Y (NPY) and agouti-related peptide (AgRP), or the anorectic transcripts proopiomelanocortin (POMC) and cocaine-amphetamine related transcript (CART). NPY and AgRP are two potent orexigenic peptides found predominantly in the ARC and whose expression and secretions are mediated through impairments in energy balance such as depletions of body fat stores, reductions in circulating glucose and/or altered metabolic hormone signaling in the brain (White and Kershaw, 1990; Kalra et al., 1991; Wilding et al., 1993; Schwartz et al., 2000). POMC and CART are also major regulators of energy balance, however, in contrast to NPY and AgRP, they produce a potent and long-lasting anorexigenic effect (Kristensen et al., 1998; Hagan et al., 1999; Larsen et al., 2000; Cowley et al., 2001). The expression and secretions of POMC and CART are mediated by signals of an energy surplus such as overfeeding and increases in body weight (Hagan et al., 1999). NPY/AgRP and POMC/CART neurons are profoundly interconnected and differentially regulated by other hypothalamic nuclei involved in energy homeostasis (Broberger et al., 1997; Parker and Herzog, 1999). Recently, it has been shown that selective activation of NPY/AgRP neurons in the ARC causes a potent suppression of POMC/CART neuronal activity, and a subsequent increase in long-term food intake (Atasoy et al., 2012). Thus, in addition to being influenced by peripheral signals indicating energy availability, NPY/AgRP neurons are capable of directly regulating activity of other neuronal subpopulations involved in food intake (Wahlestedt et al., 1986; Broberger et al., 1997; Parker and Herzog, 1999; Cowley et al., 2001; Atasoy et al., 2012). Furthermore, satiety centers such as the VMH have been shown to send excitatory inputs onto POMC/CART neurons, providing a more complex inhibition of food intake (Sternson et al., 2005). Interestingly, the excitatory glutamanergic neurons in the VMH express CRF receptors (Chalmers et al., 1995) and are thus stimulated by stress-induced CRF secretion, as well as the structurally related urocortin peptides (Chen et al., 2010; Kuperman et al., 2010). Once stimulated, these neurons project from the VMH to arcuate POMC/CART neurons and therefore initiate an anorectic signaling cascade downstream of the VMH (Chen et al., 2010). In contrast, NPY/AgRP neurons are regulated by weak inhibitory inputs from within the ARC but do not respond to the VMH (Sternson et al., 2005) and are not controlled by anorectic signals.

Peripheral signals ascending via the third ventricle and/or the vagus nerve are capable of directly and/or indirectly updating the first order arcuate neurons with information pertaining to the organism's nutritional status. Once stimulated, these first order NPY/AgRP and POMC/CART neurons project to second order hypothalamic centers located predominantly in the PVN, LH, and brain stem nuclei (Clark et al., 1984; Ramos et al., 2005; Sternson et al., 2005; Palmiter, 2007; Atasoy et al., 2012; Wu et al., 2012) to regulate meal frequency (Marin Bivens et al., 1998) and meal size, respectively (Kalra et al., 1988). NPY/AgRP neurons, for example, project directly to the PVN where they are capable of producing prolonged inhibitory post-synaptic currents and a robust feeding bout (Atasoy et al., 2012). In addition, NPY/AgRP neurons project to the LH where they serve to promote the secretion of other orexigenic transcripts such as hypocretin/orexin in response to hunger signals (Cone et al., 2001). POMC/CART neurons, on the other hand, are capable of down regulating activity in these same areas. POMC/CART project to the LH and PVN to reduce food intake and increase energy expenditure, respectively (Cone et al., 2001; Cowley et al., 2001).

### Stress and autonomic function

Integration of circulating metabolic and stress signals also occurs in several brain stem nuclei that are part of the autonomic and enteric nervous system (Berthoud et al., 2006). As such, these nuclei are critical for the responses that follow the activation of the stress axis by psychogenic, metabolic, or immunological challenges (Smith and Vale, 2006). Within the brain stem, the nucleus of the solitary tract (NTS) is the main target for information ascending from the gut via the vagus nerve and the enteric nervous system. The NTS contains noradrenergic neurons that modulate the activity of hypothalamic and limbic structures implicated in feeding, including the PVN and central nucleus of the amygdala (CeA) (Treece et al., 2000; Grill and Kaplan, 2001, 2002). Another brain stem region important for the regulation of food intake and stress is the area postrema (AP). The AP lies outside of the blood brain barrier and is sensitive to blood born nutritional signals including hormones like leptin, insulin, cholecystokinin (CCK) and ghrelin, as well as being sensitive to changes in circulating levels of glucose. Signals are relayed from the AP to the NTS where they are integrated with ascending visceral signals, as well as to the PVN where they may elicit feeding and autonomic responses (Ellenberger and Feldman, 1990; Woulfe et al., 1990; Ferguson, 1991; Castaneyra-Perdomo et al., 1992; Diaz-Regueira and Anadon, 1992; Cai et al., 1996). In addition to the regulation of feeding responses, the brain stem plays a key role in the generation of the autonomic responses, including increases in blood pressure, respiration, hepatic glucose production, vasoconstriction, lipolysis, and thermogenesis (Bamshad et al., 1998; Krukoff, 1998; Dunn et al., 2004).

Given these roles, it is not surprising that noradrenergic cells in the brain stem are affected by chronic stress. Indeed, many of the effects of chronic stress on this system are controlled by medications that block noradrenergic receptors (i.e., β-blockers) (Grassi, 2007). Moreover, NPY released from sympathetic cells into peripheral circulation may be critical for the adipogenic effects of stress, primarily by acting directly on NPY-Y2 receptors located on adipocytes (Kuo et al., 2007).

### Stress and comfort foods: The role of reward

The midbrain contains a number of cell groups implicated in brain functions underlying reward seeking, learning and memory, affective states and the generation of locomotor responses. The ventral tegmentum area (VTA) has re-emerged as an important structure in the modulation of food intake (Simerly, 2006). Dopamine (DA) cells within this region project to the striatum, prefrontal cortex, hippocampus and amygdala (Margolis et al., 2006; Fields et al., 2007; Robinson et al., 2007) where they produce behaviors to get rewards such as food and sex, or behaviors to escape aversive stimuli (Wang et al., 2004; Wise, 2004, 2006; Volkow and Wise, 2005; Baler and Volkow, 2006). These cells also play a key role in the development of addiction (Wise, 2004, 2006; Volkow and Wise, 2005). Interestingly, fasting increases DA tone and re-feeding produces an increase of DA release into the nucleus accumbens (Wang et al., 2002). Anticipation of food and other rewards also produce DA release from the VTA into the nucleus accumbens (Blackburn et al., 1992; Richardson and Gratton, 1996). In addition to DA-related processes, serotonergic components of the raphe nucleus have been implicated in the regulation of food intake (Heisler et al., 2003; Halford et al., 2007). Indeed, there are serotonin (5-HT) projections to hypothalamic and limbic centers involved in the regulation of food intake (Heym and Gladfelter, 1982; Vertes et al., 1999; Brown and Molliver, 2000; Silva et al., 2002; Jankowski and Sesack, 2004). Several lines of evidence demonstrate that 5-HT has an inhibitory effect on food intake, including clinical and experimental data on the anorectic effects of 5-HT reuptake inhibitors (i.e., SSRIs) (Halford et al., 2007). The effects of 5-HT on food intake and metabolic function are mediated, at least in part, through the stimulation of POMC cells in the hypothalamic ARC, as well as through direct effects of 5-HT onto cells of the PVN (Heisler et al., 2003).

Stress influences these midbrain regions, and chronic stress produces long lasting changes in the monoaminergic neurotransmitters produced and released by cells in these structures (Anisman et al., 2008). The way in which these systems are changed by chronic stress are numerous and may include inflammatory responses, direct and indirect glucocorticoid action and a number of associative processes that enhance the salience of external stimuli related to the stressor (Anisman and Matheson, 2005; Anisman et al., 2008). Nevertheless, stimulation of DA and 5-HT seem to be counter regulatory responses to the stress and appear to promote behaviors associated with coping including the consumption of palatable comfort foods to reduce stress (Dallman et al., 2006).

### Stress and metabolic hormones interact to regulate energy balance

In response to stress, there are several peptides and hormones released both centrally and peripherally that are capable of acting on many hypothalamic centers, which will inevitably dictate the feeding behaviors generated in response to a stressor. There are countless transmitters, peptides and hormones released directly, or indirectly, in response to stressful stimuli (See Anisman and Matheson, 2005 or Black, 2006 for reviews). Here, we will review a subset of these messengers and discuss briefly how they relate to feeding responses following stress.

#### Glucocorticoids

Both human and rodent obesity, with an emphasis on visceral obesity, have been associated with increased HPA axis activity and subsequent rises in glucocorticoid concentrations that can affect both the brain and peripheral tissues (Weaver et al., 1993). Excess production of glucocorticoids, as seen in patients with Cushing's disease for example, leads to increases in central adiposity and additional metabolic complications (Bjorntorp and Rosmond, 1999, 2000). Like other steroid hormones, the lipophilic glucocorticoids gain access to the brain via the blood brain barrier (Castonguay, 1991) and influence the expression of hypothalamic peptides involved in the regulation of food intake and energy homeostasis (Cavagnini et al., 2000; Savontaus et al., 2002). Expression of glucocorticoid receptors (GR's) has been shown in many brain regions heavily implicated in energy homeostasis such as the ARC, LH and PVN (Morimoto et al., 1996). Given that glucocorticoids are capable of regulating behaviors that control energy input and energy expenditure, they are regarded as a key factor in the link between stress and obesity. To strengthen this association, it has been shown that bilateral adrenalectomy in rats decreases glucocorticoid concentrations as well as food intake (Germano et al., 2007), and glucocorticoid replacement in adrenalectomized animals restores food intake (Green et al., 1992; Jacobson, 1999). However, the association between glucocorticoid concentration and obesity may not be as straightforward as it was once thought to be. Glucocorticoids have been shown to stimulate expression, inhibit expression and interact with, other metabolically active hormones (e.g., insulin, leptin, CRF, and others), in turn providing a much more complex control over feeding behaviors (Brindley and Rolland, 1989; Cavagnini et al., 2000; La Fleur, 2006; Jahng et al., 2008). As previously mentioned, all stressors possess the ability to elicit glucocorticoid secretion. No two stressors, however, elicit the same response from the HPA axis or the same feeding response. The degree to which the HPA axis becomes activated, and thus the amount of glucocorticoids produced in response to the stressor, depends in large part on the length and severity of the stressor at hand (Marti et al., 1994; Harris et al., 1998; Valles et al., 2000).

#### Ghrelin

Despite glucocorticoids being considered the primary hormonal culprit behind the development of stress-induced metabolic disturbances, other metabolically activate hormones may also play a role in this process. One of these potential hormones is the gut derived hormone ghrelin. Ghrelin is a 28 amino acid hormone peptide produced in the X/A-like cells of the gastric oxyntic mucosa lining the stomach (Dornonville De La Cour et al., 2001). Acutely, ghrelin has the unique ability to promote food intake (Nakazato et al., 2001; Currie et al., 2005; Date et al., 2006) an effect that is mediated by the stimulation of distinct hypothalamic, brain stem, and midbrain nuclei (Cowley et al., 2003; Seoane et al., 2003; Abizaid et al., 2006). While there are countless neuropeptides that carry out anorexic functions, to date, ghrelin is the only known gut-brain peptide that encourages food intake. In addition, ghrelin contributes to the control of long-term energy homeostasis by regulating body weight and adiposity presumably by reducing lipid oxidation (Tschop et al., 2000; Choi et al., 2003; Theander-Carrillo et al., 2006; Patterson et al., 2013).

The only known ghrelin receptor, growth hormone secretagogue receptor-1a (GHSR-1a) is widely expressed in both rodents and humans, with the highest expression found in the hypothalamus and the pituitary gland, both are areas heavily invested in energy homeostasis and the physiological response to stress. Interestingly, plasma ghrelin levels have been shown to rise in parallel with glucocorticoids in response to both acute and chronic stress, and ghrelin levels remain elevated for an extended period of time after the cessation of the stressor (Asakawa et al., 2001; Kristenssson et al., 2006; Lutter et al., 2008b; Ochi et al., 2008; Patterson et al., 2010, 2013; Chuang et al., 2011). For example, acute water avoidance stress in rats has been shown to elicit an 85% increase in circulating ghrelin 1 h after cessation of the stressor (Kristenssson et al., 2006). Similarly, 24 h after the cessation of a 2-week chronic unpredictable stress paradigm stressed animals show a significant increase in both CORT and plasma acylated ghrelin compared to non-stressed animals (Patterson et al., 2010). Finally, chronic social defeat stress has been shown to increase circulating acylated ghrelin levels (Lutter et al., 2008b; Chuang et al., 2011; Patterson et al., 2013), a change that persists up to 30 days after the stress paradigm has been terminated (Lutter et al., 2008b). Administration of ghrelin, both centrally and peripherally, has been shown to indirectly stimulate hypothalamic CRF neurons, leading to HPA axis activation (Cabral et al., 2012). Ghrelin deficient mice show a reduction in the number of CRF expressing cells in the PVN and demonstrate a hyporesponsive HPA axis in response to acute stress (Spencer et al., 2012). Interruption of ghrelin signaling, both genetically and pharmacologically, during times of chronic stress can improve the metabolic outcome of these animals suggesting that ghrelin does in fact contribute to the development of stress-induced metabolic disturbances (Lutter et al., 2008b; Chuang et al., 2011; Patterson et al., 2013). Although it is generally accepted that ghrelin stimulates food intake in response to a stressor, it remains unclear what type of food is encouraged following a stress-induced secretion of ghrelin. The caloric content of foods ingested in response to a stressor will ultimately shape the metabolic phenotype of that organism. Data from our laboratory suggests that ghrelin promotes the intake of carbohydrates while simultaneously decreasing the intake of a high-fat diet, an effect thought to reflect an acute need for rapid energy resources (Patterson et al., 2013). This idea originates from findings that stressed mice display a shift toward preferentially breaking down carbohydrates as a fuel source, while protecting adipose tissue, in a ghrelin-dependent manner (Patterson et al., 2013). In contrast, others have demonstrated ghrelin-mediated increases in high-fat diet consumption, along with a stronger conditioned place preference toward a high-fat diet, following a chronic stressor (Chuang et al., 2011). Differences between these data may reflect differences in diet availability during the stress paradigm as well as differences in the time in which the food was presented relative to the stressor itself. Thus, mice may engage in reward-based eating of comfort foods and develop a conditioned place preference to such foods after being stressed (Chuang et al., 2011), however, this may not be the case if they are tested during the stress paradigms, as is the case in our laboratory where they show a marked preference toward a carbohydrate rich diet (Patterson et al., 2013). Furthermore, an association between the presentation of the high-fat diet and the onset of the social stressor may contribute the lack of high-fat consumption seen in wild-type animals (Patterson et al., 2013).

#### Leptin

Leptin is another peripherally derived hormone, expressed in accordance to the size and amounts of adipocytes that is known to exert some control of feeding behaviors and energy homeostasis. Once secreted from peripheral adipocytes, leptin acts on hypothalamic centers (e.g., ARC) to reduce food intake and increase energy expenditure, thus encouraging a negative energy balance (Friedman, 2002). It has been suggested that leptin is also involved in the metabolic consequences of stress. Circulating glucocorticoids, for example, increase leptin mRNA expression and its secretion from adipocytes (Hardie et al., 1996; Russell et al., 1998; Williams et al., 2000). This pattern of direct glucocorticoid-mediated stimulation of leptin secretion has been shown in both humans and rodents (Miell et al., 1996; Mostyn et al., 2001). Once secreted, leptin directly stimulates anorectic POMC/CART neurons in the ARC and mediates the ability of other metabolically active hormones like ghrelin, for example, to depolarize these neurons (Elias et al., 1998; Cowley et al., 2001). Furthermore, it has been shown that leptin shares an overlapping physiological and intracellular signaling cascade with hormones such as insulin (Niswender and Schwartz, 2003) thus exerting control of feeding circuits from multiple standpoints. There is, however, contradicting data regarding interactions with leptin and glucocorticoids thus making it difficult to interpret the effects of leptin in the context of stress-induced feeding and/or changes in body weight. One group has shown that IP administration of glucocorticoids (mimicking levels seen following an acute stressor) increases leptin concentrations and therefore resulted in a decrease in food intake and body weight (Zakrzewska et al., 1999a). However, the same group demonstrated an opposing role for leptin when glucocorticoids were administered centrally, wherein NPY expression was increased and so too were food intake and body weight (Zakrzewska et al., 1999b). It remains unclear how leptin contributes to stress induced metabolic disorders and more studies are required in order to dissociate the interactions between central vs. peripheral leptin and glucocorticoids in response to stressful stimuli.

#### Others

In addition to corticosterone, ghrelin and leptin, there are a host of hormones that are stimulated by stress and that contribute directly to the regulation of food intake and energy balance. For instance, cytokines, both inflammatory and anti-inflammatory, are secreted following acute stressors (Black, 2006; Anisman et al., 2008). Chronic stressors, however, result in high levels of cytokines like IL-6 and TNF-α, both of which may contribute to obesity by causing peripheral and central leptin resistance (Velloso et al., 2008). Furthermore, while insulin may not be directly related to the stress response, its interactions with glucocorticoids allow it to participate in shaping an organism's phenotype in response to stress. For example, in the presence of insulin glucocorticoids shift their focus from mobilization of lipids to lipid accumulation (Ottosson et al., 2000). In additions, the interaction between insulin and elevated levels of glucocorticoids has been linked to increased obesity following overconsumption of comfort foods (Dallman et al., 2005). For a more detailed review on the neurochemical control of food intake see (Maniam and Morris, 2012).

## Stress models

There are many disorders/diseases that are studied through the use of animal models, and obesity is no different. Over the past century, many different stress models have been designed, some in attempt to study the effects of stress on depression or anxiety, and others to directly study the effects of stress on food intake and obesity. While it is well-documented that stress is associated with changes in body weight in rodents (Barnett, 1958; Barnett et al., 1960; Marti et al., 1994) it is not always the case that animal models mimic human reality and the basis of dissimilar findings remains poorly understood. Here, we will examine several different stress models used in the context of obesity and discuss the benefits and pitfalls of each.

### Visible burrow system

The Visible Burrow System (VBS) was originally developed as a naturalistic social stressor in attempt to investigate agonistic behaviors in rats (Blanchard et al., 1977, 1995). This model reproduces an underground burrow system, which is thought to mimic the natural habitat of a rat. Traditionally, four adult male rats, and two adult female rats, are group housed in the VBS and a social hierarchy is rapidly formed within days yielding one dominant and three submissive male animals (Tamashiro et al., 2006). This provides a very unique scenario for scientists interested in studying aggressive behaviors in rodents in a “naturalistic” setting wherein there is little requirement for intervention by the experimenter. Since its inception, others who are more interested in studying the neuroendocrine systems involved with stress, feeding and energy homeostasis have adopted the VBS. Inside the VBS, submissive animals show increased basal CORT (Blanchard et al., 1993, 1995; McKittrick et al., 1995, 2000), increases in testosterone (Blanchard et al., 1995; Hardy et al., 2002; Tamashiro et al., 2004) and reliably lose ~10–15% of their body weight (Blanchard et al., 1995; Hardy et al., 2002; Tamashiro et al., 2004). This reduction in body weight has been carefully attributed to decreases in food intake and changes in metabolic rate, while other factors such as basal locomotor activity have been eliminated as contributing factors (Tamashiro et al., 2004, 2006). The molecular underpinnings of such a robust and reliable reduction of food intake are not entirely clear. The VBS has been shown to elicit no change in the expression of orexigenic peptides or hormones, but rather elicits a reduction of leptin and insulin in submissive animals. More experiments are required for a detailed description of changes in hypothalamic feedings circuits that underlie the metabolic outcome of this stress paradigm. As a result of decreased food intake and changes in metabolic rate, submissive animals in the VBS show reductions of adiposity and a decline in lean body mass (Tamashiro et al., 2004). Interestingly, if the animals are removed from the VBS and allowed to recover, in attempt to correct their metabolic phenotype, they show a restoration of most physiological and endocrine measures, but will not regain body weight to levels equivalent to dominant or non-stressed control animals (Tamashiro et al., 2006). Animals immediately become hyperphagic and it has been shown that the gradual accumulation of body weight during a 3 week recovery from the VBS is the result of increased visceral adipose tissue (Tamashiro et al., 2006). Interestingly, the animals display persistent anhedonia and will consume less sucrose during the recovery period, compared to controls (Tamashiro et al., 2007a).

In this regard, it would appear that the VBS is not an ideal stress paradigm for the study of obesity. While there are certainly some well-documented increases in adipose tissue observed in submissive animals, this is a relative measurement to where the animals were following the participation in the VBS. In absolute terms, these animals show an overall reduction of food intake, body weight and visceral adipose tissue and therefore do not mimic human cases of stress-induced obesity. Perhaps these findings are of no surprise given the endocrine profile of these animals. Increased testosterone, for example, as seen in submissive animals has been shown to predict reduced adipose tissue (Krotkiewski et al., 1980). Interestingly, in the absence of females inside the VBS, submissive animals do not show any changes in body weight (Tamashiro et al., 2007a). It is possible that competition for resources (food, water, enrichment, females, etc.) results in submissive animals lacking energetic resources required to develop an obese-like phenotype.

### Resident intruder

The resident intruder paradigm, also known as social defeat stress, has long been used as a method for studying the effects of social dominance and subordination in animals (Ginsburg and Allee, 1942; Miczek, 1979; Miczek et al., 1982). Social hierarchies result in the expression of dominant and submissive behavioral and neuroendocrine patterns that can be used to study the effects of chronic stressors. When compared to other types of stress paradigms such as foot shock or restraint stress, the resident intruder paradigm appears to elicit the most robust physiological response, as measured by changes in hormonal and cardiovascular function (Koolhaas et al., 1997; Sgoifo et al., 1999; Keeney et al., 2006). The territorial nature of rodents allows experimenters to manipulate social hierarchies in attempt to study the physiological consequences of chronic social stressors. When an animal is single housed for ~1 week, there is a rapid development of territorial behavior within the home cage and this animal is said to be the resident (Bartolomucci et al., 2001). Traditionally, in the resident intruder paradigm, the experimenter presents a novel, non-littermate rodent to the resident inside the home cage. The novel animal is thus considered an intruder and will have to fight for his (typically performed in males only) position in the social hierarchy within that cage. It has been shown that housing animals with littermates, while permitting the formation of a social hierarchy, has no influence on CORT production or anxiety-like behaviors in submissive animals and is thus considered non-stressful (Bartolomucci et al., 2001). In contrast, when housed with a novel, non-sibling rodent, submissive animals show signs of stress such as decreased immune responses (Bartolomucci et al., 2001), increased food intake, increased body weight and fat mass (Haller et al., 1999; Bhatnagar and Vining, 2003; Foster et al., 2006; Patterson et al., 2013). It is therefore necessary to use non-familiar animals when attempting to elicit metabolic changes as a consequence of social defeat stress.

Normally, this paradigm consists of two male rodents, one resident and one intruder. There are several variants of the resident intruder paradigm, all of which reflect differences in predictability, habituation, intensity and ultimately the consequent phenotype. Typically, the intruder is presented in the home cage of the resident and they are allowed to interact until one animal subdues the other. Following interaction, animals are separated by a central divider hemisecting the home cage of the resident (Bartolomucci et al., 2001; Patterson et al., 2013). This divider permits the exchange of sensory information but forbids any physical contact between the animals. By lifting this divider and allowing the two animals to interact, the experiment presents an opportunity for the animals to establish a social hierarchy, creating four possible groups: resident-dominants, resident-subordinates, intruder-dominant and intruder-subordinate (Bartolomucci et al., 2001). The physiological outcome in animals exposed to the resident intruder paradigm seems highly dependent on the nature of the social hierarchy, in particular the change in position within the social hierarchy (Bartolomucci et al., 2001, 2004, 2005; Solomon et al., 2007). Reversal of social status in mice, for example, seen when a dominant resident becomes submissive to the intruder has been associated with immunosuppression (Devoino et al., 2003) and dramatic increases in body weight (Bartolomucci et al., 2004), while residents who did not lose their dominant status showed no changes in immune function (Devoino et al., 2003) and in some cases show reductions in body weight (Bartolomucci et al., 2004). The emergence of aggressive and dominant behavior in non-defeated intruders is considered an immunostimulant (Devoino et al., 2003) and these animals show resistance to the development of obesity, as well as predictable allocation of adipose depots (Solomon et al., 2007). Therefore, status in the social hierarchy within the resident-intruder paradigm, as seen in the VBS, can be used to predict the metabolic outcome of this stress paradigm and can even predict the location wherein animals will store adipose tissue (Solomon et al., 2007). In the context of chronic social defeat stress, some laboratories choose novel residents each day of defeat (Bhatnagar and Vining, 2003; Devoino et al., 2003; Foster et al., 2006; Keeney et al., 2006; Krishnan et al., 2007; Lutter et al., 2008a,b; Chuang et al., 2011). By employing novel residents, the experimental subjects are faced with a new social hierarchy within each unique episode of defeat. In contrast, other laboratories reuse the resident, thus maintaining an exclusive social hierarchy for the duration of the experiment (Bartolomucci et al., 2001, 2004, 2010; Moles et al., 2006; Solomon et al., 2007; Patterson et al., 2013). The former prevents habituation through predictability and the latter leads to eventual habituation and is thus considered a milder form of chronic social defeat.

Interestingly, the metabolic phenotypes generated in concert with positions in social hierarchies can be substantiated by the hormonal profile of the animals. Submissive animals in the resident intruder paradigm, for example show elevations in CORT and blood glucose, but also increases in circulating orexigenic/adipogenic plasma ghrelin (Lutter et al., 2008b; Patterson et al., 2013) and increased expression of hypothalamic NPY and AgRP (Patterson et al., 2013). Increases in ghrelin signaling leading to subsequent increases in hypothalamic NPY/AgRP results in stimulation of food intake and adiposity, with an emphasis on visceral adiposity leading to obesity. Interruption in ghrelin signaling, via pharmacological blockage or genetic manipulation, can blunt the stress-induced increases of food intake, body weight gain and adiposity seen in submissive animals (Patterson et al., 2013). Furthermore, hormonal changes that influence metabolic phenotype may vary across time throughout a chronic social defeat stress paradigms. Some physiological changes, for example, are seen immediately following an episode of defeat (Bhatnagar and Vining, 2003; Bartolomucci et al., 2004, 2005; Foster et al., 2006; Keeney et al., 2006; Lutter et al., 2008b) whereas others do not emerge until the subject has been removed from the stress paradigm and allowed to recover (Keeney et al., 2006; Moles et al., 2006; Solomon et al., 2007; Lutter et al., 2008b; Chuang et al., 2011; Patterson et al., 2013).

There are several advantages to using the resident intruder paradigm in attempt to study the physiological consequences of chronic stress. One distinct advantage is there has been a clear dissociation between food intake and body weight changes, where stressed animas will show distinct changes in body weight relative to non-stressed controls with no changes in food intake, thus suggesting that stress-induced changes in body weight and body composition are not solely attributed to increases or decreases in food intake (Haller et al., 1999; Bhatnagar and Vining, 2003; Bartolomucci et al., 2004; Moles et al., 2006; Solomon et al., 2007). Some groups have taken this one step further and dissociated the social stress aspect from the physical stress aspect of an episode of defeat and shown that social stress alone can produce changes in caloric intake (Moles et al., 2006). Interestingly, these changes in metabolism appear to extend beyond the last social interaction between resident and intruder. Subordinate animals in the resident intruder paradigm, for example, continue to show increases in body mass even weeks after last social defeat (Solomon et al., 2007; Patterson et al., 2013), when levels of food intake were equivalent to non-stressed controls (Solomon et al., 2007) suggesting that this stress paradigm left an imprint on the metabolism of these animals. This increase in body mass has been attributed to the ghrelin-dependent shift in carbohydrate utilization resulting in an increase in adipose visceral adipose tissue (Bartolomucci et al., 2001, 2004; Patterson et al., 2013) and increase overall lipid content (Moles et al., 2006; Solomon et al., 2007).

Another advantage to using the resident intruder paradigm is its apparent external validity. It has been well-documented in human cases that a higher position on a social hierarchy (as measured by socioeconomic status, for example) is associated with decreased reported stress levels (Cohen et al., 2006; Sujoldzic and De Lucia, 2007; Burdette and Hill, 2008), healthier diets (McEwen, 1998; Cartwright et al., 2003; Burdette and Hill, 2008), decreased body mass index (BMI) (George et al., 2005; Brunner et al., 2007; Burdette and Hill, 2008), etc. On the contrary, being lower on the socioeconomic hierarchy has been associated with increased stress and increased BMI (Hellerstedt and Jeffery, 1997; Laitinen et al., 2002; George et al., 2005; Cohen et al., 2006), thus shaping the trends in obesity such that humans are likely to be healthier and more fit then those beneath them on the socioeconomic ladder, regardless of their starting position. While the VBS also involves a similar type of stressor, one major difference between these two paradigms is food availability. Because a divider in the resident intruder paradigm separates the animals, they are granted unimpeded access to *ad libitum* calories, as is seen in human cases of chronic social stress.

Finally, while some stressors have anorectic effects, this particular stress paradigm causes submissive animals to overeat (Patterson et al., 2013). Overall, animals placed in the resident intruder paradigm eat more foods that are high in fat relative to non-stressed controls, but subordinate animals show increases in high fat diet intake relative to their dominant counterparts (Moles et al., 2006). However, it should be noted that the consumption of a high fat diet as a consequence of chronic stress varies from one study to another and may depend on other factors such as diet availability (time and location), gender and animal strain.

While acute social defeat is considered to be a potent stressor by means of HPA axis activity (ACTH, CORT, immune function, etc.) within hours of a defeat episode (Koolhaas et al., 1997; Sgoifo et al., 1999; Keeney et al., 2006) chronic social defeat stress results in significant habituation (Keeney et al., 2006; Patterson et al., 2013) wherein defeat stress elicits a bi-phasic response from the HPA axis. Within the first 24 h, a dramatic increase in CORT is evident following an episode of defeat. If the paradigm is continued, the rise in CORT in response to an episode of defeat is blunted for the next 2 weeks, but will show another spike if the paradigm is continued beyond 2 weeks (Keeney et al., 2006). Furthermore, there is a discrepancy in food intake, hypothalamic CRF expression and glucocorticoid secretion between acute vs. chronically defeated animals (Keeney et al., 2006; Moles et al., 2006; Patterson et al., 2013). These data are indicative of an adaptive stress response, whose ability to downplay the potency of the stressor is limited and may become detrimental in response to chronic social defeat (Keeney et al., 2006).

### Chronic mild stressors

Chronic mild stress (CMS) has been well-documented for its ability to produce depression and the wide range of accompanying physical, behavioral and neurochemical changes that accompany depression (e.g., Anhedonia) (Willner et al., 1992; Willner, 1997; Stohr et al., 2000). Generally speaking, chronic mild stress is composed of daily stressors generated randomly from a select list of possible stressors, however, CMS protocols can vary substantially from one laboratory to the next. Daily stressors could be housing the animal in a small cage or a cage with wet bedding, housing in an overcrowded cage, cage tilt, foot shock, tail pinch, changes to light/dark cycle (24 h of light, for example), housing in a soiled cage, predator odor, loud noises and more (Willner, 1991, 1997; Muscat and Willner, 1992; Willner et al., 1992; Stohr et al., 2000; Duncko et al., 2001; Westenbroek et al., 2003; Patterson et al., 2010).

The most notable physiological consequence of CMS is the induction of anhedonia, however, this model is also capable of producing impairments in conditioned place preference, reductions in brain stimulation reward, decreased sexual behavior, compromised immune function and irregular sleep architecture (Willner, 1997). These consequences of CMS are reminiscent of the physiological and neurochemical/neuroendocrine changes observed in human cases of depression, and many consequences of CMS can be corrected with *chronic* anti-depressant administration (Willner et al., 1992; Moreau et al., 1994). Because chronic, but not acute, treatment with generic antidepressants can consistently reduce the consequences of CMS, it is considered one of the best animal models of depression.

However, very few studies that use CMS elicit an obese like state in their experimental animals. Instead, it has been shown that CMS elicits decreases in the consumption of highly palatable foods (Muscat and Willner, 1992; Patterson et al., 2010) and subsequently body weight (Muscat and Willner, 1992; Westenbroek et al., 2003; Patterson et al., 2010). It is difficult to map out the hormonal and neurochemical responses as a consequence of CMS due to the inherent variability of stress paradigms. Within any given CMS protocol exists multiple forms of stressors, each capable of eliciting a unique feeding response. Furthermore, it is difficult to draw similarities between animal models of CMS and any type of human chronic stress. As previously mentioned, any given stressor poses a distinct physiological challenge to an organism and therefore elicits a distinct response. One must consider what type of CMS can be applied to a human scenario before attempting to model human obesity.

### Physical stressors

Despite the growing body of evidence suggesting that psychological stress is one of the largest sources of human stress and may in fact contribute to the obesity epidemic, most animals models aimed at investigating the role of stress on metabolic processes focus on physical, rather then psychosocial, stressors (Tamashiro et al., 2006). Physical stressors are often used in tandem with other forms of stressors in chronic stress models (e.g., UCMS) or, more frequently, as acute stressors on their own.

#### Tail pinch/foot shock

Tail pinch and foot shock, for example, are two physical stressors used commonly and have both been shown to have an impact on feeding behaviors in rodents. It was shown very early on that chronic tail pinch in rats is capable of eliciting the consumption of an energy rich diet (Rowland and Antelman, 1976). In this particular experiment the rats were encouraged to consume calories through the presence of a highly palatable, energy rich diet. It is unclear if tail pinch is sufficient enough to induce physiological changes capable of influencing dietary habits. Rather, it has been suggested that these feeding responses are a product of general arousal as opposed to an induction of motivated feeding behaviors (Greeno and Wing, 1994). Furthermore, it has been shown that an increase in feeding behavior is not observed in all subjects exposed to tail pinch, and is therefore thought to produce unreliable feeding responses in rodents (Levine and Morley, 1981).

Foot shock, on the other hand, has been shown to increase circulating plasma glucose, insulin and leptin in rats (Farias-Silva et al., 2002; Solomon et al., 2007) suggesting an anorectic hormonal profile. The hyperglycemia and hyperleptinemia observed in animals exposed to foot shock has been attributed to insulin subsensitivity in adipose tissue (Farias-Silva et al., 2002). Despite the anorectic hormonal profile in response to repeated foot shock, animals show no change in food intake but still maintain a positive energy balance and show greater cumulative weight gain relative to non-stressed controls (Solomon et al., 2007). The changes in body mass and body composition following repeated foot shocks are said to be reminiscent of the metabolic consequences of subordinate animals in the intruder resident paradigm, albeit occurred at a much faster rate (Solomon et al., 2007).

#### Restraint stress/immobilization

Physical restraint and immobilization are other commonly used methods of acute physical stressors. Restraint stress is regarded as a form of mild stress, as indicated by physiologic and neuroendocrine responses, however, it has been shown to increase expression of orexigenic peptides in the hypothalamus nonetheless (Chagra et al., 2011). Interestingly, the expression of orexigenic peptides seems to increase when restraint stress is repeated over time (Chagra et al., 2011) and it has been shown that feeding responses to restraint stress are maintained in the absence of the stressor (Tamashiro et al., 2007a). Mild restraint stress has the ability to increase the number of AgRP-expressing neurons, while simultaneously reducing α-MSH receptors, in the ARC (Chagra et al., 2011). The pattern of hypothalamic activation seems to change with repeated exposure to restraint stress. Specifically, cFos activation in the PVN, ARC and LH can be observed following acute restraint, but activation is subsided with repeated exposures suggesting habituation to this stress paradigm (Chagra et al., 2011).

Exposure to an immobilization stress, on the other hand, elicits a dramatic increase in CORT secretion and is considered to be one of the most potent stressors with respect to HPA axis hyperactivity (Haque et al., 2012). Following immobilization stress, rats have been shown to decrease their food-intake and body weight (Haque et al., 2012; Wang et al., 2012), an indication of the stressors potency. Similarly, immobilization stress has been shown to increase anorectic leptin and leptin receptor (ob-R) expression in the rat hypothalamus, particularly the ARC (Wang et al., 2012). Furthermore, no changes in NPY expression are observed following immobilization stress (Wang et al., 2012), providing hormonal data to support the feeding responses to this type of stress paradigm. It is unclear as to whether chronic exposure to immobilization stress exaggerates or blunts the feeding responses seen following acute exposure. There exists conflicting data pertaining to the potential for immobilization stress habituation (Marquez et al., 2002; Dal-Zotto et al., 2004; Wang et al., 2012).

#### Cold stress

Exposure to cold (cold stress) has been used in both rodent and human models of stress. In humans, a cold stress produces a significant rise in cortisol levels at about 15 min following the onset of the stressor (Geliebter et al., 2012). Normally, in stress protocols that are capable of eliciting an obese like phenotype, cortisol secretion can be correlated to subsequent caloric intake. However, the cortisol secretion observed in human patients following a cold stress do not correlate with subsequent caloric intake and these subjects show an overall decrease in the amount of calories consumed following the stressor relative to non-stressed controls (Geliebter et al., 2012). In contrast, cold stress in rodents has been shown to increase CORT secretion and CORT secretion was directly correlated to the upregulation of hypothalamic orexigenic peptides (e.g., NPY/AgRP) (Kuo et al., 2007). When cold stress was combined with the presence of a high-fat diet, there appears to be a significant increase in NPY and its receptor (Y2R) in white adipose tissue (Kuo et al., 2007). Furthermore, these changes were thought to elicit proliferation and differentiation of adipocytes, ultimately producing obesity and a metabolic syndrome-like condition (Kuo et al., 2007). Similar increases in Y2R in white adipose tissue have been shown following subordination stress (Patterson et al., 2013), however, it remains unclear if changes in NPY circuitry in response to cold stress are an attempt to insulate/thermoregulate or if they are due to marked changes in metabolism leading to an obese like state.

Given that cold stress can be consistently applied to both human and animal experimental subjects, but produce opposing metabolic outcomes, it is not considered to be a good model for studying stress-induced obesity. Decreasing body temperature, as can be expected during a cold stress paradigm, is metabolically expensive to an organism, as autonomic processes will immediately attempt to thermoregulate. Thermoregulation is metabolically expensive and might therefore elicit a feeding response to replenish the organisms energy stores. This in itself can be considered a major confound when attempting to study stress-induced obesity. Further studies are required to better define the role of orexigenic transcripts in response to cold stress.

## Factors influencing metabolic outcome

One hurdle faced by those using animal models to study stress-induced obesity is choosing the right model, but also the right parameters. One of the biggest difficulties when comparing results between laboratories is that their stress paradigms are often associated with different parameters (predictability, presence/absence of a calorically dense diet, presence/absence of a recovery period, time of day, etc.) Undoubtedly, there are many variables that can be manipulated by an experimenter when using any of the aforementioned stress paradigms, all of which appear to influence the metabolic outcome of the stress paradigm.

### Predictability

Predictability of a stressor will occur when experimental routines are repeated multiple times, at the same time of day in accordance with the light/dark schedule. It is well-known that the stress response adapts quickly to the presence of a stressor (McEwen, 1998, 2004; McEwen and Wingfield, 2010), and it has therefore been suggested that unpredictability of stressors will exaggerate the normal physiological response to that stressor. Intermittent bouts of social defeat, for example, have been shown to elicit very large CORT responses (Koolhaas et al., 1997; Sgoifo et al., 1999; Moles et al., 2006) and greater increases in adipose tissue deposition in rodents when compared to repeated bouts in chronic social defeat (Foster et al., 2006). Repeated bouts, on the other hand, become habituated to and elicit a dampened HPA axis response and metabolic adaptations (Keeney et al., 2006; Patterson et al., 2013). By varying the time lapse between aggressive episodes in the resident-intruder paradigm, habituation of the physiological response to the stressor cannot occur. If sustained for an extended period of time, this response may overwhelm the homeostatic mechanisms and increase the potency of that stress paradigm.

Similarly, intermittent, or acute, foot shock has been shown to cause a significant reduction in food intake and body weight, as do most physical stressors. Interestingly, when chronic foot shocks are delivered, animals are able to predict their presence, and they show no changes in caloric consumption or body weight (Griffiths et al., 1992), again lending the experimenter control over the metabolic outcome through predictability.

One must consider the evolutionary purpose of the physiological response to stress when discussing how predictability influences the outcome of a particular stressor. The physiological responses to stress are generated in attempt to meet the energetic demands of the particular stressor, liberate the appropriate energy resources while temporarily down regulating any non-pertinent bodily functions. As previously discussed, the acute stress response is an adaptive response and is one that is necessary for the survival and well-being of an organism. However, this may not be the case when dealing with protracted stressors and even more so to unpredictable protracted stressors. Habituation of the CORT response and other secreted hormones may be indicative that the demand for energy in response to the stressor may also have familiarized, thus avoiding any unnecessary changes to metabolism. However, if the animal cannot predict the presence of the stressor, then metabolic habituation is not possible which exerts the largest expense on the organism and ultimately produces the greatest metabolic consequence.

### Intensity

The intensity of a stressor can be gauged through several objective measures such as glucocorticoid secretion (i.e., CORT), circulating CRF/ACTH levels, cFos activation in stress related brain regions, etc. There are limited number of studies investigating the relationship between stress severity and metabolic outcome, however, as a rule of thumb, the magnitude of food intake reduction as a consequence of stress can be used as a general marker of stress severity. For example, in an experiment wherein 3 types of physical stressors were used to represent low, medium and high intensity (handling, restraint, and immobilization) there was a graduated anorexic response as the intensity of the stressors increased (Marti et al., 1994). Interestingly, in this particular experiment they also employed a restraint stress (medium intensity) for different durations of time and showed no differences in reactivity, suggesting that it is in fact dependent on the stressor itself and not the length of time it is administered (Marti et al., 1994). The same pattern has been shown when comparing feeding responses between a mild food shock (1.5 mA) and a potent immobilization stressor, where immobilization leads to a significantly greater reduction in food intake, body weight and increased CORT secretion (Rabasa et al., 2011). Similar findings have been reported from human cases, wherein eating patterns of US Marine's are negatively correlated with severity of stress exposure (Popper et al., 1989). Stress severity not only influences feeding response during time of stress, but it may also predict the persistent feeding behaviors during the recovery period (Harris et al., 1998; Valles et al., 2000). This suggests that stress severity, even in acute stressors, may in fact shape metabolic outcome of stress paradigm for the long-term.

Physiologically speaking, what makes one stressor more potent then the next? Of course one would have to consider varying levels of glucocorticoid secretion, or other metabolically active hormones, given the extent to which each stressor activates CRF neurons in the PVN. However, this may not be the case. It has been shown that many different stress paradigms, both physical and psychological stressors, with seemingly different potencies elicit indiscriminate activation of the HPA axis, with measurable differences in amygdala activation (Dayas et al., 2001). Furthermore, it is well-documented that the melanocortin system is an integral component to the anorexic responses to stress. Interestingly MC4 receptors are densely expressed in the amygdala, and when activated, these particular receptors potently inhibit food intake (Liu et al., 2013). Therefore, perhaps analysis of regional activation, outside of the HPA axis, in areas like the amygdala can better predict the intensity of a stressor and therefore the metabolic outcome. More experiments need to be conducted to better understand this phenomena.

### Gender differences

Like many other disorders (e.g., schizophrenia, major depression, anxiety, etc.), gender seems to play a key role in determining the overarching consequences of stress on body weight management. While there exists evidence suggesting that, in humans, there is no sex difference in stress exposure (Young and Korszun, 2010), there also exists evidence suggesting that women are more likely to acknowledge and/or recognize an event as being stressful (Goldman et al., 2005). In humans, women often report more stress-related eating (Greeno and Wing, 1994) and more consumption of foods high in fats and sugars in response to stressful situations (Wansink et al., 2003) compared to men. In support of these data, it has been suggested that perhaps the feeding responses to stressors is different between men and women as a result of their coping strategies (Grunberg and Straub, 1992). Men, for example, tend to display the typical fight or flight response to a stressor while women are more likely to seek out social support following stress, a strategy termed “tend-befriend” (Taylor et al., 2000).

Once again, we look to the use of animal models to further clarify the role of gender in stress induced metabolic dysfunctions. Under basal conditions, female rats show higher levels of CORT then do males (Stohr et al., 2000) which sets the stage for divergent phenotypes in response to stress. There is an abundance of evidence demonstrating gender-specific effects of social defeat stress (Haller et al., 1999), social instability stress (Haller et al., 1999), chronic mild stress (Stohr et al., 2000; Dalla et al., 2005, 2008; Shors et al., 2007), foot shock (Iwasaki-Sekino et al., 2009) acute forced swim test and the development of learned helplessness (Shors et al., 2007; Dalla et al., 2008, 2010). There is very limited data pertaining to the effect of social stressors on females. In the context of social defeat, males seem to be more vulnerable to changes in body weight and food intake then do females (Haller et al., 1999). It is possible that the aggressive nature of males renders them more susceptible to detrimental effects of social stressors with an aggressive component. In comparison, social stressors that lack aggression (e.g., overcrowded housing or social isolation, unpredictable social environments) elicit a greater response in female rats compared to males (Haller et al., 1999; Schmidt et al., 2010).

Given that, in humans, women are twice as likely to develop major depression then men (Kendler et al., 2002; Marcus et al., 2005), the majority of animal work done investigating gender differences in response to stress have employed the use of CMS and acute physical stressors due to their superiority in modeling human depression. It has been shown that both CMS and acute foot shock elicits an exaggerated CORT response in females compared to males (Dalla et al., 2005; Iwasaki-Sekino et al., 2009), suggesting that gender affects stress-related regions of the brain. Additionally, female rats show disruption of estrous cycles, increased floating time in a forced swim test, no reduction of body weight and dramatic changes in hippocampal serotonergic and dopaminergic neurotransmission compared to male rats, in response to CMS (Dalla et al., 2005). Interestingly, these data reflect, to some extent, human conditions of depression wherein hypo-serotonergic function in the hippocampus may lead to depressive like symptoms and correction with antidepressants, whom increase serotonergic neurotransmission, can alleviate atrophy seen in hippocampal neurons. Here we see that female rats showed reduced hippocampal serotonin turnover in response to CMS, whereas males showed no changes (Dalla et al., 2005). As would be expected, these same female rats show reduced locomotion in an open field test and increased immobility in a forced swim test (Dalla et al., 2005), behaviors reminiscent of human depression.

### Genetics and genetic models

It is not surprising that genetics also contribute to the metabolic outcome of some stress paradigms. When discussing rodent models of stress, different rodent strains will reliably acquire distinct metabolic phenotypes in response to equivalent stressors. It is well-documented that different strains of rodents show different effects of social stress (Berton et al., 1998) and unpredictable chronic mild stressors including both physical and psychological components (Stohr et al., 2000; Mineur et al., 2003; Pothion et al., 2004; Yalcin et al., 2008). Under basal conditions, for example, Fischer rats show a hyperactive HPA axis, as evidenced by elevated CORT levels, compared to the hypoactive HPA axis of the Lewis rat (Stohr et al., 2000). This provides a unique opportunity for experimenters to investigate the relationship between HPA axis activity and metabolic outcome of stress paradigms. For example, it has been shown that Fischer rats are more susceptible to the anxiogenic effects of CMS compared to Lewis rats, as would be expected based on their genetic differences (Stohr et al., 2000). Fischer rats also demonstrate differences in their peak CORT response and negative feedback loops associated with the HPA axis as measured by a two fold increase in latency to return to basal CORT levels following a stressor (Stohr et al., 2000). Interestingly, in this study Lewis rats showed greater startle responses and more risk assessment behaviors as compared to Fischer rats (Stohr et al., 2000), an effect thought to be mediated by CRF.

The same phenomena has been demonstrated in different mouse strains, wherein BALB/c and C3H/He strains appear more sensitive to an unpredictable CMS compared to C57BL/6J mice, for example (Mineur et al., 2003). Similarly, differences in body weight changes, sucrose consumption, water maze performance, grooming behaviors, coat characteristics and responses to forced swim following unpredictable CMS vary significantly over different strains of mice (Mineur et al., 2003; Pothion et al., 2004; Yalcin et al., 2008). In a study by Pothion et al. (2004), it was demonstrated that sucrose preference over water, and baseline body weights, were consistent across 11 different strains of mice. When exposed to an unpredictable CMS, however, some obvious strain differences were observed wherein stressed mice consumed significantly less sucrose (CBA/H and C57 BL/6J) and gained significantly less weight (CBA/H only) relative to control mice, a phenomena that did not occur in any other strain (Pothion et al., 2004). Similarly, others have shown a reduction in sucrose consumption in CD-1, DBA/2J, C57 BL/6J and BALB/C mice in response to acute footshock while this effect was entirely absent in A/J mice (Griffiths et al., 1992). Furthermore, the extent of sucrose consumption decrease varied across different strains of mice where CD-1, DBA/2J and BALB/C mice showed a greater reduction compared to that of C57BL/6J and A/J mice (Griffiths et al., 1992).

In addition to comparing how different rodent strains respond to a variety of stress paradigms, many have used knock-out models, wherein the gene encoding a specific peptide or receptor has been removed, to explore the roles and contributions of these peptide signaling systems in response to stress. The use of ghrelin and ghrelin receptor knockout mice has been instrumental in determining the role of central ghrelin in mediating the orexigenic, adipogenic and anxiolytic effects of ghrelin following chronic social defeat stress (Lutter et al., 2008b; Patterson et al., 2013). Others have used the well-defined leptin deficient ob/ob mouse model to determine leptin's role in stress-induced feeding and anxiety. The obese-like phenotype in ob/ob mice is characterized by hypercorticosteronemia and leptin injections given to ob/ob mice reverse this hormonal profile (Ahima et al., 1996) suggesting that leptin plays a key role in the regulation of the HPA axis. It should be of no surprise that knock-out models that interrupt normal ghrelin and/or leptin signaling will influence the metabolic response to a stressor given the dense expression of ghrelin and leptin receptors on neurons containing NPY/AgRP, POMC/CART, CRF, ACTH and more. Similarly, urocortin knock-out animals have been used to help characterize the role or urocortins in mediating the physiological response to stress. Urocortin-2 deficient mice, for example, show elevations in basal stress hormones (i.e., ACTH) and do not show a rebound feeding in response to a 2 h caloric restriction test (Chen et al., 2006). Furthermore, urocortin-2 deficient mice display an antidepressant-like phenotype when exposed to both a forced swim test and a tail-suspension test (Chen et al., 2006). CRF2 receptor deficient mice also show premature inactivation of the HPA axis (Coste et al., 2000), both illustrating urocortin's influence on HPA axis activation as well as stress induced food intake and anxiety (Coste et al., 2000; Chen et al., 2006). The same patterns of diverse physiological responses can be expected from other rodent models wherein the normal expression of hypothalamic peptides is interrupted genetically.

Interestingly, the responses to antidepressants can also vary across different rodent strains and genetically modified rodent models (Yalcin et al., 2008) suggesting not only a genetic component to stress susceptibility but to the treatment of stress-related disorders as well. For example, it has been shown that hypothalamic orexigenic peptides such as ghrelin and orexin mediate the depressive-like symptoms and antidepressant-like effects of calorie restriction following chronic stress (Lutter et al., 2008a,b). These findings are relevant given the association between stress, depression and feeding. Calorically restricted orexin-null mice, for example, show anxiety like behaviors following chronic social defeat where wild-type littermates do not (Lutter et al., 2008a). Similarly ghrelin is capable of dampening the depressive like symptoms that normally arise following chronic social defeat (Lutter et al., 2008b). These data highlight the ability of hypothalamic feeding circuitry to influence the physiological response to stress and alter the behavioral and metabolic responses generated.

## Conclusion

It has been well-established that stressful environments are conducive to the development of obesity and related metabolic disorders. Indeed, the stress response represents a metabolic challenge that is followed by physiological and behavioral responses in attempt to meet this challenge. Evolutionary pressures have shaped these homeostatic and allostatic responses, yet continued exposure to stressors and the constant struggle to meet these energetic demands may ultimately serve as detrimental. Chronic stress may therefore alter the response to stressors and tilt the energy homeostatic scale toward an increase in energy consumption and adiposity. A detailed understanding of the interaction between the stress response, the nature of the stressors and their impact on food intake and energy balance regulation may allow for more personalized treatments of obesity, be it pharmacologically or psychologically based. The use of animal models will certainly increase our understanding of these types of interactions and aid in defining the neurobiological underpinnings of consequent metabolic disorders. However, a comprehensive understanding of the wide array of stress paradigms poses its own challenges. Using variants, or derivatives, of established stress paradigms makes any type of direct comparison of data between laboratories difficult. As a result, many different stressors are broadly categorized (e.g., Table 1) and may not reflect the physiological differences between them. Perhaps an attempt can be made to better distinguish the details surrounding these stress paradigms or implement some type of standardization. One thing is for certain, however, in that many different stressors pose distinctive energetic challenges on an organism, and while metabolic processes are adjusted to compensate for these challenges, many result in the conservation of adipose tissue.

**Table 1:** **Summary of stress paradigms, hormonal responses and physiological outcome**.

**Stress paradigm**	**Duration**	**Species**	**Intensity**	**Predictability**	**Physiological response**	**Food intake/body composition**	**Reference(s)**
Visual burrow system	Chronic (3 weeks)	Long evans rats	^+^	^+++^	Increased CORT^1^/decreased CORT^2^, reduced leptin and insulin^2^	Decrease in food intake resulting in 10–15% body weight reduction	^1^Blanchard et al., 1995
							^2^Tamashiro et al., 2004
Resident intruder paradigm	Intermittent	Syrian hamsters	^+++^	^+^	Increased leptin	Increased food intake, body weight and adiposity	Foster et al., 2006
Resident intruder paradigm	^1^Chronic(10 days)	^1^C57 BL6/J Mice	^+++^	^+^	Increases in CORT, ghrelin^1,3^, NPY^3^, AgRP^3^	Increased food intake; no change in body weight^1^; Increased carbohydrate intake, body weight and adiposity^2,3^	^1^Lutter et al., 2008a,b
	^2,3^Chronic(21 days)	^2^CD-1 Mice	^++^	^+++^			^2^Bartolomucci et al., 2004, 2009
		^3^C57 BL6/J Mice	^++^	^+++^			^3^Patterson et al., 2013
Chronic mild stress	Chronic	^1^Lister hooded rats	^++^	^+^	Increase CORT^1,2^ and ghrelin^2^,	Anhedonia and depression^1^;	^1^Muscat and Willner, 1992
		^2^C57 BL6/J-DBA Mice				Decreased consumption of palatable diet and body weight^1,2^	^2^Patterson et al., 2010
Foot shock	Acute (1 day)	Syrian hamsters	^++^	^+^	Increased leptin, no change in insulin	Increased body mass, feed efficiency and adipose tissue	Solomon et al., 2007
Foot shock	Chronic (3–14 days)	^1^Wistar rats	^++^	^++^	^1^Increased glucose, increased insulin	^1^Increased lipolysis	^1^Farias-Silva et al., 2002;
		^2^C57 BL6/J Mice				^2^Decrease in caloric consumption; no change in body weight	^2^Griffiths et al., 1992
		^2^BALB/c Mice				
Tail pinch	Chronic (6^.^days^−1^ × 5 days)	Sprague–Dawley rats	^+^	^+++^	n/a	Increased caloric intake and body weight	Rowland and Antelman, 1976
Restraint	^1^Acute (1 day)	^1^Sprague–Dawley rats	^+^	^+^	Increase cFOS in PVN^1,2^, LH^1^ and ARC^1^;	Orexigenic effects^1^	^1^Chagra et al., 2011
	^2^Acute (15 min)	^2^C57 BL6/J Mice				Anxiogenic effects^2^	^2^Spencer et al., 2012
					Increased CORT^2^		
Restraint	Chronic (14 days)	Sprague–Dawley rats	^+^	^+++^	Increased LH AgRP;	Increased feeding-related behaviors and redistribution of energy stores	Chagra et al., 2011
					Decreased MC4-R in ARC and LH		
Immobilization	Acute (2 h)	Albino wistar rats	^+++^	^+^	Dramatic increase in CORT	Decrease food intake and body weight	Haque et al., 2012
Immobilization	^1^Chronic (1 h × 7 days)	Sprague–Dawley rats	^+++^	^+^	Dramatic increase in CORT; increase in leptin^2^; no changes in NPY^2^	Decrease food intake and body weight	^1^Rabasa et al., 2011
		^2^Chronic (3 h × 21 days)					^2^Wang et al., 2012
Cold stress	Chronic (14 days–3 months)	C57 BL6/J mice	^+^	^+++^	Increase CORT and NPY/AgRP, Y2R	Increased adiposity	Kuo et al., 2007

+ , Mild;

++ , Moderate;

+++ *, High*.

### Conflict of interest statement

The authors declare that the research was conducted in the absence of any commercial or financial relationships that could be construed as a potential conflict of interest.
